# LV-pIN-KDEL: a novel lentiviral vector demonstrates the morphology, dynamics and continuity of the endoplasmic reticulum in live neurones

**DOI:** 10.1186/1471-2202-9-10

**Published:** 2008-01-23

**Authors:** Vicky C Jones, Lynn McKeown, Alexei Verkhratsky, Owen T Jones

**Affiliations:** 1 Faculty of Life Sciences, The University of Manchester, Manchester, M13 9NT, UK; 2 Institute of Experimental Medicine, ASCR, Videnska 1083, 142 20 Prague 4, Czech Republic

## Abstract

**Background:**

The neuronal endoplasmic reticulum (ER) is an extensive, complex endomembrane system, containing Ca^2+ ^pumps, and Ca^2+ ^channels that permit it to act as a dynamic calcium store. Currently, there is controversy over the continuity of the ER in neurones, how this intersects with calcium signalling and the possibility of physical compartmentalisation. Unfortunately, available probes of ER structure such as vital dyes are limited by their membrane specificity. The introduction of ER-targeted GFP plasmids has been a considerable step forward, but these are difficult to express in neurones through conventional transfection approaches. To circumvent such problems we have engineered a novel ER-targeted GFP construct, termed pIN-KDEL, into a 3^rd ^generation replication-defective, self-inactivating lentiviral vector system capable of mediating gene transduction in diverse dividing and post-mitotic mammalian cells, including neurones.

**Results:**

Following its expression in HEK293 (or COS-7) cells, LV-pIN-KDEL yielded a pattern of fluorescence that co-localised exclusively with the ER marker sec61β but with no other major organelle. We found no evidence for cytotoxicity and only rarely inclusion body formation. To explore the utility of the probe in resolving the ER in live cells, HEK293 or COS-7 cells were transduced with LV-pIN-KDEL and, after 48 h, imaged directly at intervals from 1 min to several hours. LV-pIN-KDEL fluorescence revealed the endoplasmic reticulum as a tubular lattice structure whose morphology can change markedly within seconds. Although GFP can be phototoxic, the integrity of the cells and ER was retained for several weeks and even after light exposure for periods up to 24 h. Using LV-pIN-KDEL we have imaged the ER in diverse fixed neuronal cultures and, using real-time imaging, found evidence for extensive, dynamic remodelling of the neuronal ER in live hippocampal cultures, brain slices, explants and glia. Finally, through a Fluorescence Loss in Photobleaching (FLIP) approach, continuous irradiation at a single region of interest removed all the fluorescence of LV-pIN-KDEL-transduced nerve cells in explant cultures, thus, providing compelling evidence that in neurons the endoplasmic reticulum is not only dynamic but also continuous.

**Conclusion:**

The lentiviral-based ER-targeted reporter, LV-pIN-KDEL, offers considerable advantages over present systems for defining the architecture of the ER, especially in primary cells such as neurones that are notoriously difficult to transfect. Images and continuous photobleaching experiments of LV-pIN-KDEL-transduced neurones demonstrate that the endoplasmic reticulum is a dynamic structure with a single continuous lumen. The introduction of LV-pIN-KDEL is anticipated to greatly facilitate a real-time visualisation of the structural plasticity and continuous nature of the neuronal ER in healthy and diseased brain tissue.

## Background

Since its early recognition as a 'lace-like' intracellular organelle [1], the endoplasmic reticulum (ER) has emerged as a structure of pivotal importance in eukaryotic cell function [2]. The ER mediates lipid biosynthesis and, by acting as the entry point of nascent proteins into the secretory pathway, controls the insertion of polypeptides destined for secretion [3]. Further, ER-resident enzymatic systems control the topology, assembly and early glycosylation of integral proteins destined for intracellular organelles and the cell surface [2]. In addition to its biosynthetic functions, the ER also plays a key role in cell signalling by acting as a regulated store of calcium ions [4]. In healthy cells, intra-luminal Ca^2+ ^levels regulate a plethora of spatiotemporally distinct vital cellular processes including intracellular Ca^2+ ^signalling and protein folding within the ER [5]. Any disruption of the homeostatic mechanisms governing intra-luminal ER Ca^2+ ^levels has serious pathophysiological consequences and can often trigger cell death [6-9].

In spite of the cardinal role of the ER in cell physiology, the mechanisms underlying its structural and functional plasticity are not fully understood. In live cells, the ER is a tubulovesicular structure extending in 3-dimensions from the inner nuclear membrane to the cell cortex [10]. The ER morphology is highly dynamic [11], not least because of the budding of transport vesicles from specialised transitional (tER) sites [12] exiting to the Golgi apparatus and their fusion upon returning to the ER [13]. Consequently, mechanisms must exist which enable the ER to sustain its signalling functions in spite of ongoing changes in morphology [12]. Evidence showing that the ER contains a single continuous lumen argues for the potential diffusion of various molecules throughout the entire ER network [4,14-17]. However, some functional studies suggest that Ca^2+ ^signalling may involve structurally segregated Ca^2+ ^stores in a discontinuous ER network [18]. One cell type where the resolution of such issues is especially important is the neurone. In neurones, the ER is present in the soma and throughout the axons and dendrites [19-21]. In addition to supporting often localised protein synthesis [22], the neuronal ER is thought to regulate synaptic plasticity and integrative physiology in a manner that is likely to depend critically on ER morphology [23]. For example, in central neurones, where the dendritic ER extends into many, but not all, dendritic spines [24], the degree of continuity in the ER lumen would determine the spread of post-synaptic Ca^2+ ^signals between adjacent spines and possibly over much longer distances to the soma [25-27].

From the above, a definition of the architecture of the ER in live cells, including neurones, is of paramount importance in determining the ability of the ER to act as a signalling organelle and whether its possible fragmentation [9] is a (patho)physiological process that shapes Ca^2+ ^signalling [4,28]. Unfortunately, present methods to define the architecture of the ER have severe limitations. Thus, immunocytochemical and thin-section electron microscopic methods are cell-destructive and limited by the volume of tissue that can feasibly be analysed [14,20,21,24,29].

The introduction of lipophilic vital dyes such as diIC16 has facilitated live imaging [11], but many are non-specific, cytotoxic or unsuitable for long-term studies. Moreover, as noted previously [21], the use of such dyes relies upon their diffusion in the plane of the lipid bilayer. Thus, they can only access ER membranes which are by definition continuous, leaving open the possibility that discontinuous tracts of ER may go unlabeled. A major step forward has been the introduction of vectors capable of expressing genes encoding ER-targeted green fluorescent protein [7,30]. Such vectors show excellent targeting specificity to the ER lumen, minimal binding to cellular components, and suitable optical properties for quantitative photobleaching. Unfortunately, the utility of such cDNA vectors is limited by the low efficiency with which post-mitotic cells, such as neurones, can be transfected [31].

We now describe a powerful and versatile new tool for defining the architecture and dynamics of the ER in live cells, including neurones, based upon the lentivirus-mediated transduction [32,33] of a novel ER-targeted GFP reporter (LV-pIN-KDEL). Through fluorescence imaging, we show robust and specific expression of LV-pIN-KDEL in the ER of simple cell lines, astrocytes and neurones in both dissociated culture and in live brain slices and explants. Moreover, using a Fluorescence Loss in Photobleaching (FLIP), strategy, we demonstrate that the neuronal endoplasmic reticulum is continuous in LV-pIN-KDEL-transduced explant cultures.

## Results

The development of a system to dissect the dynamics of the neuronal ER was achieved in two stages. First, the construction of a versatile reporter for the faithful visualisation of the ER was achieved and second, its insertion into a 3^rd ^generation lentiviral system capable of neuronal transduction. Constraints guiding construction of the reporter included requirements for (i) a cleavable signal peptide for its targeting into the lumen of the ER, for (ii) a Green Fluorescent Protein for visualisation in live cells and (iii) a motif for concentration of the reporter in the ER lumen. Although such constructs are available commercially, most suffer from requirements for intron splicing, lack of cloning sites and epitope tags for alternative modes of detection [30]. For these reasons we, therefore, designed an entirely novel reporter based upon the trafficking probe pIN-G – which we described recently [34].

**Figure 1:**
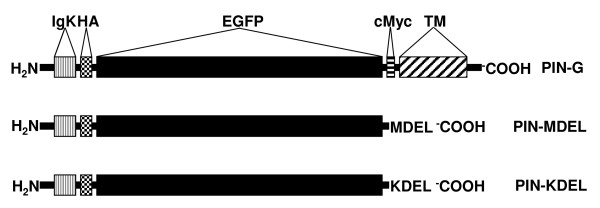
**Comparison of the features encoded by pIN-KDEL, pIN-MDEL and pIN-G expression vectors**. Following expression and cleavage of the Igk-chain leader sequence, pIN-G protein adopts a transmembrane orientation where HA and cMyc epitope tags and GFP have an intra-lumenal disposition in the ER and trafficking intermediates of the secretory pathway, (but extracellular at the cell surface) [34]. In contrast, the derivatives pIN-MDEL and pIN-KDEL, which lack a transmembrane (TM) anchor, are expressed as soluble proteins in the ER lumen. While pIN-MDEL escapes the ER and enters the anterograde secretory pathway, pIN-KDEL is retained in the ER by virtue of its carboxy-terminal KDEL motif.

The pIN-G construct encodes an artificial type I membrane protein containing a signal peptide, GFP and HA and cMyc epitope tags (Fig. 1). Upon inspection of the pIN-G sequence we noted a region 3' to GFP that, upon mutation at position 1455 to a stop codon (Tyr-*; TAC:TAG), would be predicted to cause premature truncation of pIN-G to yield a soluble, HA-tagged, GFP, lacking the c-Myc and transmembrane domains of pIN-G (termed pIN-MDEL). More important, pIN-MDEL is predicted to contain a carboxy-teminal Met-Asp-Glu-Leu (MDEL) sequence that could readily be mutated to the carboxy-terminal Lys-Asp-Glu-Leu (KDEL) motif that serves to retrieve ER-resident proteins once they have reached the early Golgi-network [13,35]. Using site-directed mutagenesis we, therefore, first converted pIN-G to pIN-MDEL and then mutated the methionine codon (position 1442) of pIN-MDEL to a lysine residue (Met:Lys; ATG:AAG). The resulting construct – pIN-KDEL – was then tested for its ability to target the endoplasmic reticulum.

**Figure 2:**
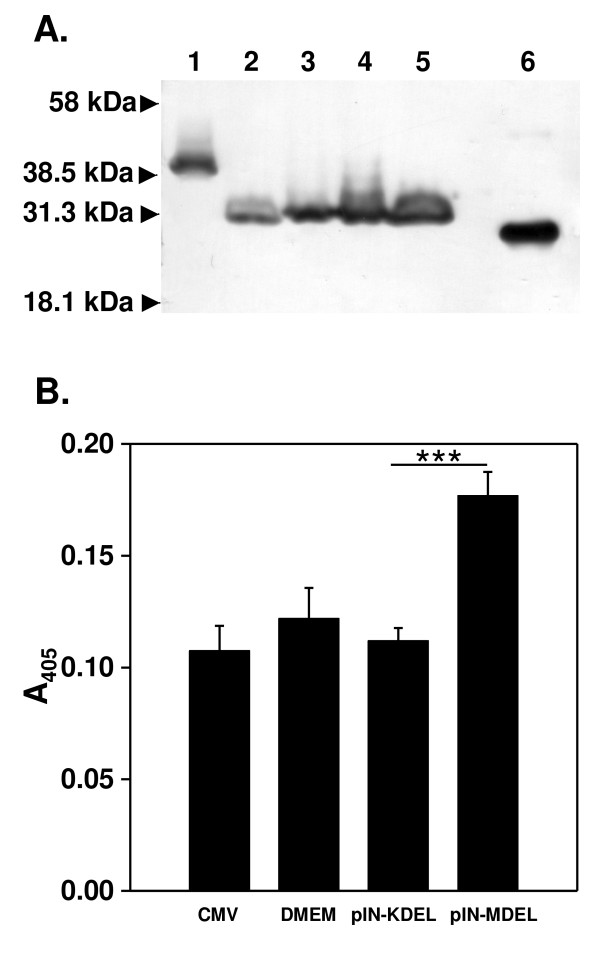
**Verification of pIN-KDEL expression and intracellular retention**. **A**. Immunoblots of lysates from transfected HEK293 cells reveal bands corresponding to the anticipated sizes for pIN-G (41.3 kDa) (Lane 1) and the smaller (31.4 kDa) pIN-MDEL (Lane 2) and pIN-KDEL (Lane 3) proteins. Identical sized proteins were obtained through transduction of HEK293 cells with a lentivirus encoding pIN-MDEL (Lane 4) and pIN-KDEL (Lane 5). A band corresponding to EGFP (Lane 6) is shown for comparison. Transfection, transduction and immunoblotting were done as described in Materials and Methods. Immunoblots were analysed using anti-GFP at 1:4000 dilution. **B**. Anti-EGFP ELISA at 48 h post-transfection reveals the presence of EGFP immunoreactivity in the culture medium from cells expressing pIN-MDEL, but not pIN-KDEL, when compared to HEK293 cultures that were mock-transfected or transfected with insert-free plasmid.

As shown in Figure 2A, immunoblots of lysates from transfected HEK293 cells revealed bands of 31.4 kDa for pIN-MDEL and pIN-KDEL, confirming truncation of the pIN-G reporter (41.5 kDa). The pIN-MDEL and pIN-KDEL bands were of higher mass than that for soluble GFP, indicating that they did not arise through partial degradation. In contrast to the KDEL motif, the MDEL sequence does not mediate ER retention. Thus, pIN-MDEL is predicted to be secreted from the cell following its traffic via the anterograde secretory pathway, while pIN-KDEL is retained intracellularly within the ER [35]. In support of this contention, anti-GFP ELISA of cell culture supernatants from HEK293 cells transfected with pIN-MDEL showed significant levels of GFP immunoreactivity, while those from pIN-KDEL transfectants were similar to either mock-transfected or non-transfected cells (Figure 2B).

Direct evidence for targeting of pIN-KDEL to the ER was obtained through deconvolution fluorescence imaging of transfected cells treated with established antibody markers (in parentheses) to membranous organelles including the endoplasmic reticulum (Sec61β)[36], the Golgi apparatus (GM130) [37], the early endosomes (EEA-1) [38] and the lysosomes (LAMP-1) [39].

**Figure 3:**
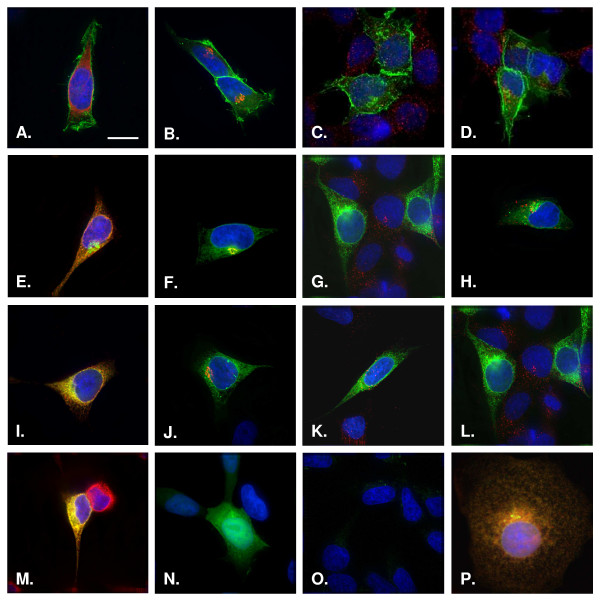
**pIN-KDEL is targeted to the endoplasmic reticulum**. HEK293 cells transiently transfected with pIN-G (panels A-D), pIN-MDEL (panels E-H) and pIN-KDEL (panels I-L, P) were fixed at 48 h, permeabilized and treated with antibodies (See Materials and Methods) to the following organelle markers: Sec61β (Endoplasmic Reticulum)(panels A, E, I, M, P); GM130 (Golgi apparatus)(panels B, F, J); EEA1 (Early endosomes)(panels C, G, K); LAMP-1 (Lysosomes) (D, H, L), followed by the appropriate Cy-3 conjugated secondary antibody. Green denotes EGFP fluorescence while red corresponds to the organelle marker. Areas of red/green overlap are shown in yellow. Blue indicates DAPI-stained nuclei. Scale Bar: 15 μM. Cells expressing pIN-KDEL (panels I-L, P) or a commercially available reporter (panel M), show strong overlapping fluorescence with the ER marker Sec61β (panels A, E, I, M). Extensive overlap with the ER is also seen with pIN-KDEL transfected COS-7 cells (panel P). In contrast, pIN-MDEL (panels E-H) and pIN-G (panels A-D) are found throughout the anterograde or entire secretory pathway, respectively. Fluorescence is present throughout the cytoplasm of cells transfected with a plasmid encoding EGFP (panel N) and is absent in cells transfected with an insert-free plasmid (panel O). Scale bar 15 μm.

As shown in Figure 3I – L, HEK293 cells transfected with pIN-KDEL yielded a pattern of fluorescence that overlapped exclusively with the ER marker sec61β (panel I), but with no other major organelles. Moreover, the pattern of expression of pIN-KDEL was identical to that seen with a commercially available ER-targeted GFP reporter (Fig 3M) [30]. Equally important, the targeting of pIN-KDEL to the ER did not appear to be cell-specific as transfection of the COS-7 monkey cell line yielded a strong pattern of ER expression (Fig 3P), whose morphology was similar to that reported previously [40].

In contrast to pIN-KDEL, pIN-G was localised throughout the secretory pathway including the ER (Fig. 3A), Golgi apparatus (Fig. 3B), early endosomes (Fig 3C) and lysosomes (Fig 3D). The control reporter pIN-MDEL was primarily localised to the ER (Fig. 3E) and Golgi apparatus (Fig. 3F). However, pIN-MDEL was absent from endosomes (Fig. 3F) and lysosomes (Fig. 3G), consistent with the notion that the presence of an Igκ leader sequence, but no additional trafficking motifs, directed it to the lumen of organelles of the anterograde secretory pathway. In cells transfected with GFP alone, fluorescence was dispersed throughout the cytoplasm (Fig. 3N). No fluorescence labelling was seen upon omission of primary antibody to mock-transfected cells (Fig. 3O). As with, pIN-G, we found no evidence for cytotoxicity or inclusion body formation [40].

**Figure 4:**
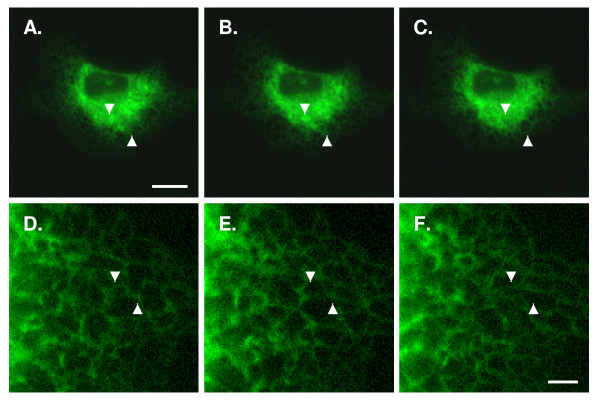
**Expression of pIN-KDEL reveals the dynamic morphology of the endoplasmic reticulum in live cells**. COS-7 cells expressing pIN-KDEL were imaged 48 h post-transfection (Materials and Methods) and subjected to sequential recording of images at a rate of 1 frame/second (Supplemental movie 1). Image frames displayed at low magnification at intervals of 0, 30 and 240 sec (panels A-C, respectively) reveal changes in the overall ER morphology within the cell (Arrowheads). At higher magnification, select frames 0, 10 and 60 sec (panels D-F, respectively) reveal rapid, detailed changes in ER morphology including tubule formation, lengthening and ring opening and closing (Arrowheads). Scale bars: A-C, 10 μm; D-F, 2 μm.

To explore the utility of pIN-KDEL in resolving the ER in live cells, COS-7 cells (similar data were obtained for HEK293; data not shown) were transfected with pIN-KDEL and, after 48 h, imaged directly at consecutive intervals ranging from 10 sec to several hours. As shown in Fig. 4 and Additional file 1, pIN-KDEL fluorescence reveals the endoplasmic reticulum of live cells to be a tubular lattice structure whose morphology can change markedly within seconds. Although GFP has been reported to be phototoxic [41], both the integrity of the cells and the ER are retained even after prolonged and repeated light exposure for periods of up to 24 h.

**Figure 5:**
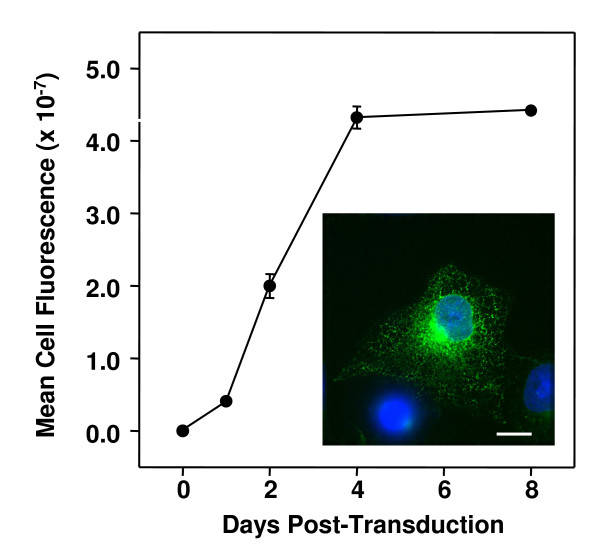
**Time-course of lentivirus-mediated pIN-KDEL expression in COS-7 cells**. Sub-confluent COS-7 cells in culture, were treated with pIN-KDEL lentiviral particles, fixed, stained with DAPI and imaged at the post-transduction times indicated. Images were analysed using Image J, by first, making maxium intensity Z projections of the green (EGFP) channel, followed by subtraction of background fluorescence defined as that <3× the average. The integrated channel fluorescence within the field was then corrected for the number of cells (determined by counting DAPI stained nuclei in the blue channel) to yield an average fluorescence/cell. Data shown are the mean and standard error for at least 10 independent fields (>5 cells) of view/day. Inset: Example of LV-pIN-KDEL (green) transduced COS-7 cell; DAPI (blue), note similarity to image obtained by transfection (Fig 3, panel P). Scale bar 15 μm.

Taken together, the data above indicate that pIN-KDEL is a useful probe for imaging the ER in both fixed and living mammalian cells. To extend the utility of the probe to visualising the ER in live neurones, pIN-KDEL was engineered into a 3^rd ^generation replication-defective, self-inactivating lentiviral gene delivery vector pseudotyped with a heterologous envelope containing protein G of the vesicular stomatitis virus, Indiana serotype (VSV.G) [32]. The lentiviral vector system has been shown to be capable of mediating gene transduction in diverse dividing and non-dividing mammalian cells, including neurones [32,42-45]. The resulting viral particle preparation, termed LV-pIN-KDEL, showed robust expression of pIN-KDEL within a few days (t_1/2 _50 h) that reached a maximum at day 4 which was sustained until day 8, post-transduction (Fig. 5). The pattern of fluorescence observed (Fig. 5, inset) was identical to that seen upon direct transfection of COS-7 cells (Fig. 3P). The efficiency of transduction, while dependent upon viral titre (typically 5 × 10^8 ^TU/ml), often reached 70–80% of the total cell population in HEK293 and COS-7 cells.

**Figure 6:**
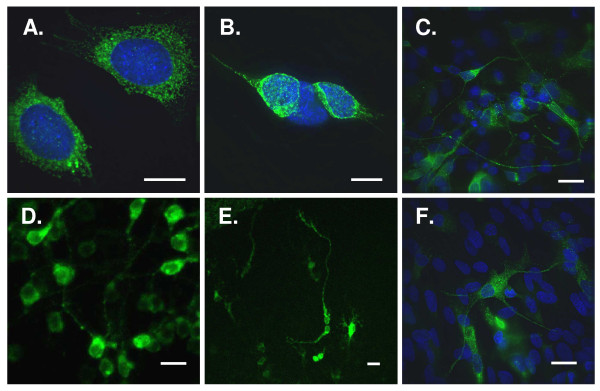
**LV-pIN-KDEL reveals the ER in diverse neuronal and non-neuronal preparations**. Lentivirus-mediated delivery of pIN-KDEL reveals the ER (green) in N38 neuroendocrine (A) and SHSY5Y (B) cell lines; neurones (C) and astrocytes (F) in dissociated rat hippocampal cultures; and cortical neurones in organotypic brain slice (D) and explant (E) cultures. Cells were fixed, in some cases stained with DAPI (nuclei), and imaged as described in Materials and Methods. Scale bars: A, B, 15 μm; C-F, 30 μm.

More significant, LV-pIN-KDEL was able to mediate transduction of a wide-range of neuronal cell types including the N38 (Fig. 6A) and SHSY5Y (Fig 6B) neuronal cell lines, hippocampal neurones in dissociated culture (Fig. 6C) and cortical neurones in organotypic (Fig. 6D) and explant (Fig. 6E) preparations. The efficiency of transduction was typically 30–70% and depended on the tissue thickness and viral titre. In brain slices and explants, transduction was most evident in supraficial layers although occasionally cells deeper in the tissue were labelled. Our data also support the contention that lentiviral preparations pseudotyped with VSV-G show some specificity for neurones [42]. However, in dissociated culture LV-pIN-KDEL expression was noted in some astroglia (panel F). In all neurones and glia examined, the ER extended throughout the cell with no evidence of marked fragmentation.

**Figure 7:**
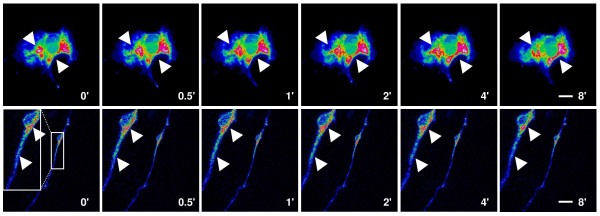
**Morphological dynamics in the endoplasmic reticulum of live astrocytes and neurones transduced with LV-pIN-KDEL**. Rat astrocytes in dissociated culture (top panels) and cortical neurones in organotypic brain slice (Bottom panels; 2.5× magnified inset) were treated with LV-pIN-KDEL and imaged at 48–72 h post transduction (Materials and Methods). Images were recorded sequentially from a single astrocyte or neurone at a rate of 2 frames per minute (Supplemental movies 2 and 3, respectively). Individual frames are shown corresponding to the indicated capture times. To facilitate the visualisation of alterations in EGFP intensity corresponding to changes in ER morphology, all images were processed using the ratio look-up table facility in Image J. Areas showing changes in ER morphology are indicated by arrowheads. Images of the neurone are shown as pairs corresponding to a low magnification frame (Scale bar: 30 μm) and at 2.5× higher magnification (inset left) of the boxed area shown at t = 0'.

We next asked whether LV-pIN-KDEL could be used to examine the morphology of the ER in live glia and neurones? In these experiments LV-pIN-KDEL-transduced astrocytes and cortical neurones were imaged directly at consecutive intervals ranging from 30 sec to 8'. To identify changes in morphology, the fluorescence intensity of each frame was presented as a ratio of the maximum image intensity. As shown in Fig 7 (top) and Additional file 2, pIN-KDEL fluorescence reveals rapid, continuous, dynamic rearrangement of the ER throughout the cell. A similar dynamic picture of ER morphology was obtained in frames captured from cortical neurones (Fig 7 (Bottom) and Additional file 3). Here, the ER was found to extend continuously from the soma into the neurites. Dynamic changes in ER structure, including thinning and coalescence into regions of high density, occurred on a timescale of seconds. In no instance did we find evidence for fragmentation of the ER, again suggesting its internal continuity in neurones.

**Figure 8:**
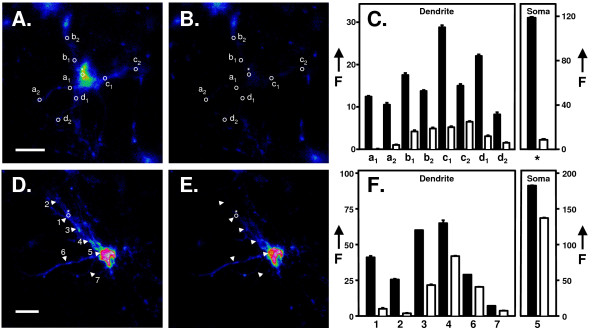
**Fluorescence loss in photobleaching (FLIP) experiments demonstrate continuity of the endoplasmic reticulum in cortical explant neurones transduced with LV-pIN-KDEL**. Rat cortical explant cultures were treated with LV-pIN-KDEL and imaged at 48–72 h post transduction (Materials and Methods). Images were recorded sequentially from transduced neurones and then subject to high intensity laser photobleaching at a region of interest in the soma (*, panel A, 4 minutes bleaching) or dendrite (panel site 1, 5 minutes bleaching). The beam was then switched to that used for imaging and images collected (panels B and E) and images collected. The fluorescence remaining in the cells was then quantified pre- (panels A and D) and post-bleach (panels B and E). Fluorescence intensity (F) data from the select regions of interest denoted in panels A and D, are shown in panels C and F, respectively. To facilitate the visualisation of alterations in EGFP intensity corresponding to changes in ER morphology, all images were processed using the ratio look-up table facility in Image J. Scale bar: 30 μm.

Finally, we asked whether the ER in neurones was comprised of a single continuous compartment using a Fluorescence Loss in Photobleaching (FLIP), strategy. Here, LV-pIN-KDEL cortical explants were imaged and then subjected to continuous irradiation at a single small (1 μm diameter) site with laser light of sufficient energy to induce photobleaching. Following the bleach period, the neurones were then re-imaged and images quantified pre and post-bleach. A comparison of images of an explant neurone pre- (Fig. 8, panel A) and post-bleaching for 4 minutes (Fig. 8, panel B) at a site on the soma (indicated by an asterisk) caused 94% loss of fluorescence (panel C). Significantly, fluorescence decreased in all other regions of the cell including both initial (a_1_-d_1_) and more distal dendritic regions (a_2_-d_2_) by 50–90%. No loss of fluorescence was seen in cells that had not undergone spot photobleaching. Thus, fluorophores located at even the furthermost regions are able to diffuse into the ER located in the irradiated spot on the soma. We next asked if fluorescence bleaching at a site on the dendrites could induce a global decrease in LV-pIN-KDEL fluorescence. A comparison of images of a different explant pre- (Fig. 8, panel D) and post-bleaching (Fig. 8, panel E) at a site on the dendrites (site 1, panel D) revealed a decrease in fluorescence in all regions of the cell (e.g. sites 2–7, Panel D). The decrease in fluorescence upon 5 minutes of photobleaching showed some variation, with regions proximal to the bleach site (e.g. site 2 and 3) experiencing greater loss than most other regions (panel F). Loss of fluorescence was greatest at sites where the dendritic arbour was thin (Sites 1,2 and 7) and lower for dendrites that were thicker (sites 4 and 6, Panels D, F). However, irrespective of their magnitude, decreases were detected throughout, including at the soma (site 5, panels D, F). Again, no loss of fluorescence was seen in cells that had not undergone spot photobleaching. Qualitatively identical results were obtained in all cells studied, (n = 6, 4 explants). Throughout, we found no evidence for tracts of fluorophores that failed to succumb to complete depletion. In cases where bleaching was incomplete, such as those where the bleaching spot was on dendrites rather than somata (e.g. Fig. 8, panels D-F), more prolonged bleach periods eventually removed >95% of the fluorescence.

## Discussion

In this paper, we have described the design, characterisation and utility of a novel lentiviral vector – LV-pIN-KDEL – for defining the morphology of the endoplasmic reticulum in live neurones in vitro and in situ. Our vector builds upon the advantages of the previously documented and commercially available GFP-based ER-targeting vectors [30], while eliminating their disadvantages. Thus, LV-pIN-KDEL and its parent pIN-KDEL vector show excellent and specific targeting of GFP to the ER lumen, but are simpler, smaller, intron-less constructs containing additional functionality by virtue of sequences encoding an HA epitope for immunocytochemistry or pull-down assays, and restriction sites for further modifications. Although somewhat demanding to prepare, the LV-pIN-KDEL pseudovirus can be made in large batches and stored indefinitely [32]. Once thawed, expression is effected simply by addition of the viral suspensions to target cell preparations. The most important feature of LV-pIN-KDEL, however, is the ease with which it is expressed in post-mitotic cells, and particularly in neurones, which are otherwise notoriously difficult to transfect.

Here we have shown that LV-pIN-KDEL is readily expressed in simple HEK293 and COS-7 cell lines, astrocytes and central neurones grown in dissociated and organotypic cell culture. Furthermore, we have also obtained expression in neurones of the dorsal root ganglion, exocrine and other cells (not shown). It is also noteworthy that lentiviral expression is possible through direct microinjection into select brain regions in whole animals [43,44]. The pleiotropy of LV-pIN-KDEL is primarily a function of the VSV-G coat with which it is pseudotyped [42,43] and the use of a strong CMV promoter. The VSV-G coat protein mediates transduction of most mammalian cell types, but pseudotyping with alternate coat proteins and the use of other promoters could readily be used to alter the patterns of cell expression [42,43].

The lentiviral gene delivery system we employed is based on a 3^rd ^generation replication-deficient system optimised for gene therapy applications. As such it shows considerable biosafety and can be used in standard level II conditions [32,46]. Although non-infectious, the LV pseudovirus does mediate stable insertion of pIN-KDEL into the genome and, thus, requires caution in handling to avoid accidental exposure by inhalation or injection [46]. Such stable integration does, have advantages, however, not least in the facile preparation of cell lines and transgenic animals.

The precise patterns of LV-pIN-KDEL expression we obtained conform to those anticipated from previous studies on ER morphology. In live COS-7 and HEK293 cells, the ER is highly dynamic, showing the sliding, ring closing and branching motions documented elsewhere [11,40,46,47]. The structure and dynamic nature of the neuronal ER also conforms to that found in previous studies [23] on hippocampal cultures.

Taken together, the FLIP data argue that a period of localised photobleaching causes a global decrease in fluorescence in the neurone that is specific to the bleached cell and is independent of the site of bleaching. The decreases in fluorescence observed are greater when bleaching is localised to the soma compared to the dendrites. This observation is in accord with the notion that the laser intersects a greater ER volume element, and, hence, bleaches more fluorophores per unit of time in somata rather than dendrites. Irrespective of the site, photobleaching causes a loss of fluorescence throughout the cell, compatible with a single continuous ER-lumen between soma and dendrites. These results, thus, fit well with recent observations on the ability of calcium signals to flux between these compartments in dopaminergic neurones [48].

## Conclusion

In summary, we have developed a lentiviral-based method – LV-pIN-KDEL – to express a novel ER-targeted fluorescent reporter in the nervous system. The lentiviral system is easy to use and affords high levels of transduction in diverse cells including simple cell lines and primary neurones in dissociated and slice culture. Using LV-pIN-KDEL, in conjunction with imaging and laser-induced photobleaching, the ER has been shown to be a highly dynamic structure with a single continuous lumen. The availability of the LV-pIN-KDEL system, and its use in conjunction with probes of physiological function should now permit us to test this possibility rigorously and dissect the interplay between neuronal ER morphology and those signalling pathways involved in nerve function.

## Methods

Parent construct pIN-G (Genbank AY841887) was designed and prepared as described recently [34]. The Lentiviral transfer construct pCCL.sin.PPT.prom.GFP.Wpre and packaging plasmids pMD2-VSV-G, pMDLg/pRRE and pRSV-REV were a gift from P.-L. Nicotera (University of Leicester, UK). Antibodies used for identifying intracellular organelles were obtained from the following sources: Anti-GM130, anti-EEA-1: BD Biosciences Pharmingen, Oxford, U.K. Anti-Sec61β was obtained from Upstate Biotechnology, UK. The Lamp-1 antibody (clone H4A3), was developed by August and Hildreth and was procured from the Developmental Studies Hybridoma Bank, Iowa, U.S.A. Immunoblotting reagents were from Invitrogen, Paisley, U.K. Oligonucleotides were synthesised by Sigma-Genosys, U.K. Site directed mutagenesis was performed using the Quikchange II XL kit (Stratagene, The Netherlands) in accordance with the manufacturer's instructions. All other chemicals were of reagent grade or higher purity.

### Construction of pIN-KDEL mammalian expression vector

Generation of an ER-targeted GFP fusion protein was achieved in two stages. First, the conversion of a transmembrane GFP-reporter construct – pIN-G – to one expressing a secreted soluble homologue – pIN-MDEL – through site-directed mutagenesis of pIN-G using the following forward and reverse oligonucleotide primers: 5'-GAC GAG CTG TAG AAG TCC GGA CTC AGA TCT-3' and 5'-GAG ATC TGA GTC CGG ACT TCT ACA GCT CGT C-3'. Second, conversion of pIN-MDEL to its ER-targeted homologue – pIN-KDEL (Fig. 1) – via site-directed mutagenesis of pIN-MDEL using the following forward and reverse oligonucleotide primers: 5'-GGG ATC ACT CTT GGC AAG GAC GAG CTG TAG-3' and 5'-CTA CAG CTC GTC CTT GCC AAG AGT GAT CCC-3'.

### Construction of pIN-KDEL lentiviral transfer vector

To generate a plasmid mediating transduction of pIN-KDEL by lentivirus (pLV-pIN-KDEL), pIN-KDEL (above) was digested with the restriction enzymes Sna BI and Sal I. The resulting 1257 bp fragment was then cloned into the corresponding sites of the lentiviral transfer construct pCCL.sin.PPT.prom.GFP.Wpre [32] using T4 ligase [49]. An additional control construct, (pLV-pIN-MDEL) was generated by site-directed mutagenesis of pLV-pIN-KDEL using the following forward and reverse primers: 5'-GGG ATC ACT CTT GGC ATG GAC GAG CTG TAG-3' and 5'-CTA CAG CTC GTC CAT GCC AAG AGT GAT CCC-3'. The identity of all constructs was confirmed by DNA sequencing (University of Manchester, Core Facility).

### Viral packaging

Third generation HIV-1 derived lentiviral vectors, pseudotyped with envelope protein G of vesicular stomatitis virus (VSV-G), were prepared through calcium phosphate-mediated transient co-transfection of HEK293-FT cells (Invitrogen) with: the self-inactivating transfer construct bearing HIV-1 cis-acting sequences and the desired transgene (above) driven by the internal promoter; a plasmid, pMD2-VSV-G, encoding a heterologous VSV-G (Indiana serotype) under control of a CMV promoter; a conditional packaging plasmid pMDLg/pRRE expressing the *gag *and *pol *genes driven by a CMV promoter and a fourth plasmid pRSV-REV expressing *rev *gene under the control of the Rous Sarcoma Virus (RSV) promoter [32]. After two days, transfected cell culture supernatants were collected, concentrated using Vivaspin 20 centrifugal concentrators (Sartorius Ltd., UK) and used immediately or stored frozen in aliquots at -80°C. All steps were performed under level II biosafety conditions.

### Determination of viral titre

The functional titre (expressed as transduction units (TU)/ml) of the viral preparations was assessed by determining the number of GFP+ve cells at 48 h following transduction of HEK293 cells (10^5^/well) at 75% confluence, with dilutions of viral stock. The specific transduction activity (infectivity) was obtained by determining the concentration of p24 capsid protein with a gag-p24 ELISA kit (Trinity Biotech, Carlsbad, CA). The infectivity was then calculated as TU/ng p24. After concentration, typical preparations had titres of up to 5 × 10^8 ^TU/ml and infectivities of around 1 × 10^5^TU/ng p24.

### Transfection and transduction

HEK293 cells were subcultured and grown at 37°C in a humidified incubator and maintained with 5% CO_2 _as described [34]. COS-7 cells were grown under identical conditions. E17 Hippocampal neurones in primary culture were obtained and grown as described [50]. Brain slices or whole cortex explants were taken from E17 rat embryos and maintained as described [51].

Transient transfections were performed using Fugene 6 (Roche, UK), in accord with the manufacturer's instructions as described [34]. Lentiviral transduction of HEK and COS7 cells was performed by adding 50 μl of the requisite viral preparation (above) to cell culture medium. After 48 h, cells were subject to live imaging or immunocytochemistry. Hippocampal preparations, brain slices and cortical explants were treated with 100 μl concentrated virus on day 3 in culture and subjected to live imaging or immunocytochemistry on day 5 – 6.

### Immunostaining of transfected HEK293 cells

Following transduction or transfection, cells were fixed in 4% paraformaldehyde for 20 minutes at room temperature, washed in phosphate buffered saline (PBS), quenched with 0.1 M glycine and mounted onto glass slides using ProLong Gold anti-fade (Molecular Probes, OR). Intracellular staining of organelles was performed by permeabilising fixed cells with 0.5% saponin in PBS for 5 minutes followed by incubation with antibodies raised against the appropriate markers at a concentration of 5–10 μg/ml in 0.01% saponin in PBS. Detection of specific staining was performed with fluorophore-conjugated secondary antibodies (Stratech Scientific Ltd., Jackson ImmunoResearch, UK) at 1:200.

### Imaging

Fixed cells, on mounted coverslips, were visualised using a Deltavision restoration deconvolution system (Applied Precision Instruments, Seattle) attached to an Olympus IX-70 inverted microscope fitted with a Photometrics CoolSNAP HQ CCD camera. Epifluorescence images were acquired using a ×100 UPLAN objective (NA 1.35) and filter sets for DAPI/FITC/Texas red and cy3/cy5, recording widefield optical sections of 0.2 μm throughout the z plane of the cells. The corresponding z stacks were then deconvoluted using a constrained iterative algorithm assigned by the manufacturer on a Unix-based computer system equipped with SoftWoRx version 3.5.0, acquisition software.

Live-cell imaging was performed using a Leica DM IRE2 inverted microscope equipped with an automatic shutter, RGB filter set, HCX PL APO ×63 37°C-corrected glycerine objective and a Roper CoolSnap HQ camera. Cells were maintained in a chamber controlled for temperature and CO_2_. Live images were captured as z-stacks or single optical planes, at specified intervals ranging between 1 and 60 s over a period of 5 min to overnight.

Imaging of living brain slices and cortical explants was performed using a Leica TCS SP5 tandem-head inverted confocal microscope with argon excitation laser, HC PL Fluotar 10× (NA 0.3) air objective and LAS AF software. Tissues were held in a temperature-controlled chamber while live images were obtained.

### Fluorescence loss in photobleaching

Live cortical explants were exposed to sustained, bleaching levels of 488 nm laser light using a live-imaging TCS Leica SP5 confocal microscope (see imaging above). To minimise the potential for simultaneously bleaching multiple, discontinuous regions of ER, the bleach region of interest (ROI), selected for each cell, was confined to a single spot of 1 μm diameter; the smallest area allowed by this microscope system. In the basic photobleaching protocol, cells were imaged with low intensity 488 nm laser light, and then the selected bleach ROI exposed to the laser at maximum power for 2–5 minutes. The laser was then switched back to the low power settings used for imaging. A comparison of mean fluorescence intensity before and after bleaching was then made for ROIs in selected areas of the cell using NIH Image J software.

## List of abbreviations

HEK293, human embryonic kidney 293; DMEM, Dulbecco's minimal essential medium; FBS, Foetal bovine serum; GFP, green fluorescent protein; PBS, phosphate-buffered saline; SDS-PAGE, sodium dodecyl sulphate polyacrylamide gel electrophoresis. FLIP, Fluorescence Loss In Photobleaching. ROI, region of interest.

## Authors' contributions

VJ carried out the design and construction of the ER-targeted pIN constructs, preparation of the lentivirus and all biochemical and imaging and FLIP assays and assisted with design of the project. LM prepared the original pIN construct, established the lentiviral delivery system and participated in the lentiviral preparations. AV helped conceive the study, participated in its design and helped draft the manuscript. OJ conceived the design of the pIN construct, the use of the lentiviral system and biochemical assays, coordination and design of the project and drafting of the manuscript. All authors read, contributed and approved the final manuscript.

## Supplementary Material

Additional file 1 Movie showing ER dynamics in live COS-7. This movie, taken at 1 frame per second, indicates the dynamic re-arrangements experienced by the ER in COS-7 cells revealed after transfection with pIN-KDEL. Click here for file

Additional file 2 Movie showing ER dynamics in live hippocampal astrocytes. This movie, taken at 2 frame per second, indicates the dynamic re-arrangements experienced by the ER in astrocytes transduced with LV-pIN-KDEL. Click here for file

Additional file 3 Movie showing ER dynamics in live hippocampal astrocytes. This movie, taken at 2 frame per second, indicates the dynamic re-arrangements experienced by the ER in astrocytes transduced with LV-pIN-KDEL. Click here for file
